# Adaptation by Type V-A and V-B CRISPR-Cas Systems Demonstrates Conserved Protospacer Selection Mechanisms Between Diverse CRISPR-Cas Types

**DOI:** 10.1089/crispr.2021.0150

**Published:** 2022-08-12

**Authors:** Wen Y. Wu, Simon A. Jackson, Cristóbal Almendros, Anna C. Haagsma, Suzan Yilmaz, Gerrit Gort, John van der Oost, Stan J.J. Brouns, Raymond H.J. Staals

**Affiliations:** ^1^ Laboratory of Microbiology, Wageningen University and Research, Wageningen, The Netherlands; Wageningen University and Research, Wageningen, The Netherlands.; ^2^ Department of Microbiology and Immunology, University of Otago, Dunedin, New Zealand; Wageningen University and Research, Wageningen, The Netherlands.; ^3^ Department of Bionanoscience, Delft University of Technology, Delft, The Netherlands; Wageningen University and Research, Wageningen, The Netherlands.; ^4^ Kavli Institute of Nanoscience, Delft, The Netherlands; and Wageningen University and Research, Wageningen, The Netherlands.; ^5^ Biometris, Wageningen University and Research, Wageningen, The Netherlands.

## Abstract

Adaptation of clustered regularly interspaced short palindromic repeats (CRISPR) arrays is a crucial process responsible for the unique, adaptive nature of CRISPR-Cas immune systems. The acquisition of new CRISPR spacers from mobile genetic elements has previously been studied for several types of CRISPR-Cas systems. In this study, we used a high-throughput sequencing approach to characterize CRISPR adaptation of the type V-A system from *Francisella novicida* and the type V-B system from *Alicyclobacillus acidoterrestris*. In contrast to other class 2 CRISPR-Cas systems, we found that for the type V-A and V-B systems, the Cas12 nucleases are dispensable for spacer acquisition, with only Cas1 and Cas2 (type V-A) or Cas4/1 and Cas2 (type V-B) being necessary and sufficient. Whereas the catalytic activity of Cas4 is not essential for adaptation, Cas4 activity is required for correct protospacer adjacent motif selection in both systems and for prespacer trimming in type V-A. In addition, we provide evidence for acquisition of RecBCD-produced DNA fragments by both systems, but with spacers derived from foreign DNA being incorporated preferentially over those derived from the host chromosome. Our work shows that several spacer acquisition mechanisms are conserved between diverse CRISPR-Cas systems, but also highlights unexpected nuances between similar systems that generally contribute to a bias of gaining immunity against invading genetic elements.

## Introduction

As a response toward mobile genetic elements (MGEs), prokaryotes have evolved a diverse arsenal of immune systems. The only known adaptive immune system is CRISPR-Cas, comprising CRISPR (clustered regularly interspaced short palindromic repeats) arrays and gene clusters that encode the CRISPR-associated Cas proteins. Genetic memories of prior invaders are stored in CRISPR arrays as short variable sequences (spacers), separated by short invariable sequences (repeats). The CRISPR arrays are transcribed as precursor crRNAs and then processed to produce mature crRNAs. These mature crRNAs guide Cas nucleases or Cas nuclease complexes to complementary target DNA or RNA sequences (protospacers). Addition of new spacers to CRISPR arrays occurs through a process termed CRISPR adaptation, which can be naive or primed.^1–5^ Naive adaptation occurs when the CRISPR array does not contain a preexisting spacer against a specific MGE, whereas primed adaptation is a positive feedback loop using targeting through existing spacers to enhance CRISPR adaptation.^2,3,6–8^

In both cases, CRISPR-Cas systems use protein-based sequence recognition of protospacer adjacent motifs (PAMs) to increase the speed of target searching, and to prevent autoimmune self-targeting of the CRISPR loci.^9–11^ As such, new spacers must be selected with appropriate PAM sequences for efficient target recognition.^12^

CRISPR adaptation in most systems is catalyzed by a spacer acquisition complex containing the core proteins Cas1 and Cas2. Some systems rely on additional Cas or host-encoded proteins. For example, the *Streptococcus pyogenes* type II-A system requires Csn2 and Cas9 for efficient adaptation.^1,13^ In addition, multiple type I, II, and V systems rely on Cas4 for PAM selection and spacer length trimming^14–16^ and some systems use reverse-transcriptase–Cas1 fusions to acquire spacers from RNA.^17–20^ To date, most CRISPR adaptation research has focused on class 1 systems, generally type I, whereas comparatively little is known about class 2 systems apart from type II-A, II-C, V-C, and VI-B.^21–24^

In this study, we elucidate the Cas protein requirements for adaptation of class 2 type V-A and V-B CRISPR-Cas systems from *Francisella novicida* and *Alicyclobacillus acidoterrestris*, respectively. Different combinations of *cas* genes (and mutants thereof) from these systems were expressed in *Escherichia coli*, and acquired spacers were detected by polymerase chain reaction (PCR) and analyzed by high-throughput sequencing. We found that Cas12 and Cas4 were not required for adaptation, but Cas4 activity was required for PAM selection in both systems and impacted spacer lengths in the type V-A system. We also discovered evidence of spacer acquisition from RecBCD-produced substrates, which parallels the mechanisms found in type I systems that favor spacer acquisition from MGEs rather than from the host.^25^ Overall, our findings demonstrate that several aspects of CRISPR adaptation previously observed in type I systems are more widely conserved.

## Methods

### Bacterial strains and growth conditions

The *E. coli* strains DH5-α and DH10-β were used for plasmid cloning. The adaptation growth experiments were performed using *E. coli* BL21-AI, which encodes T7 RNA polymerase under an arabinose-inducible promoter. Cells were grown at 37°C at 220 rpm in lysogeny broth. Where required, media were supplemented with the following: ampicillin (Ap) 100 μg/mL, spectinomycin (Sp) 100 μg/mL, and chloramphenicol (Cm) 35 μg/mL.

### Plasmid construction

For the CRISPR adaptation experiments in *E. coli*, we used the following three plasmids (or variants thereof): pAdaptation, pEffector, and pTarget. These plasmids encode spectinomycin, chloramphenicol, and ampicillin resistance and use compatible CloDF13, p15A, and pMB1 origins of replication, respectively. Primers, plasmids, and *cas* gene mutations used in this study are listed in Supplementary Tables S1–S3, respectively. Detailed cloning strategy of all plasmids can be found in Supplementary Table S4. The initial pAdaptation plasmids (pCas4_1_2_VA_pre1 and pCas4/1_2_pre1) were cloned by ligation-independent cloning. pCas4_1_2_VA_pre1 did not encode a short N-terminal Cas4 sequence due to misannotation of the start codon of *cas4* on the genome. This N-terminal sequence was later added through PCR to create pCas4_1_2_VA_pre2. Variants of the pAdaptation plasmids were created by digestion and ligation (Supplementary Table S4). pCas_4(I-G)/1_2 was constructed by Gibson assembly (NEBuilder HiFi DNA Assembly Master Mix).

A multiple sequence alignment of Cas4 homologs led us to identify mutations in *cas4 and cas4/1* in *F. novicida* U112 and *A. acidoterrestris* ATCC 49025, respectively, which result in N-terminal truncations that are not conserved in other strains (Supplementary Fig. S1). Therefore, we reverted these mutations, by site-directed mutagenesis to restore the full Cas4 sequences. For the pEffector plasmids, pCas12a was constructed by PCR amplification of pACYC-duet-Cas12a-Cas4_1_2 followed by self-ligation of the amplified product. pCas12 was used to construct pCas12a(RuvC) and pCas12a(PI) by restriction digestion and ligation with inserts obtained from pRham_Cas12a(RuvC) and pRham_Cas12a(PI), respectively. pCas12b was constructed by restriction digestion and ligation of *cas12b* into pACYC-duet. pCas12b(RuvC) was constructed by Gibson assembly. pCas12b(PI) was constructed using GoldenGate assembly.

After further inspection, the *cas12b* cloned included a short native sequence on the N-terminal end, which was then removed by around the horn PCR so that only the *cas12b* coding sequence was present in the plasmid. pTarget was constructed by Gibson assembly with parts obtained from p2A-T (Addgene No. 29665) and pUA66. Protospacers for targeting and priming were introduced by inverse PCR amplification of the plasmid using primers containing the protospacer in the overhang.

### Adaptation growth experiment

Competent BL21-AI cells containing pAdaptation and pEffector were transformed with the pTarget variants (pNaive, pTargeted, or pPriming), then plated on agar plates containing Ap, Sp, and Cm, and incubated overnight at 37°C. The following day, three colonies from each plate were inoculated into 2 mL of medium in 15-mL Falcon tubes. Cells were grown for 3 h at 37°C (shaking), and then, the *cas* gene expression was induced by the addition of 2 g/L l-arabinose and 0.5 mM of isopropyl β-d-1-thiogalactopyranoside. After an additional 48 h of growth, the culture densities (OD_600_) were measured and the cultures were adjusted to an OD_600_ of 1. Cells from 200 μL samples of each OD-corrected culture were harvested by centrifugation at 16,000 *g* for 1 min, then resuspended in 50 μL Milli-Q water, and stored at −20°C.

### Population PCR

For population PCRs to detect acquired spacers, 2 μL of OD_600_-normalized cells were used as template in 50 μL reactions using the Q5 High-Fidelity 2 × Master Mix (New England Biolabs) and with forward primers that matched the repeat plus a single 3′ nucleotide mismatch with the first base of the existing spacer. This PCR strategy allows preferential amplification of expanded compared with unexpanded arrays but with the trade-off of not detecting new spacers beginning with the same nucleotide as the existing spacer.^26,27^

To reduce removal of the 3′ degenerate base by exonuclease activity of the Q5 DNA polymerase, which would result in loss of primer specificity for expanded arrays, we included a phosphorothioate bond before the 3′ degenerate base; we observed that PCRs using primers without this phosphorothioate modification were less specific for expanded versus nonexpanded arrays (Supplementary Fig. S2). PCRs and thermocycling conditions were carried out according to the manufacturer's protocol. Initial denaturation at 95°C was 10 min, extension time 30 s, and the annealing temperature was 67°C and 70°C for V-A and V-B, respectively. Amplicons were separated by gel electrophoresis using 3% agarose gels.

### Illumina MiSeq sequencing of spacer acquisition

To prepare the samples for high-throughput sequencing, PCR products were purified by spin-column clean-up, and then quantified using a Qubit dsDNA broad range assay. Equal molar amounts of product from each sample were pooled into two libraries, keeping the type V-A and V-B samples separate. Sequencing libraries were prepared with the NEB Next Ultra II DNA Library Prep Kit and the NEBNext Multiplex Oligo Index primer set 1, according to the manufacturer's protocol. Libraries were pooled equally and spiked with ∼20% of PhiX control library (Illumina), then sequenced using an Illumina MiSeq v3 flow cell (2 × 300 base paired-end). Image analysis, base calling, demultiplexing, and data quality assessments were performed on the MiSeq instrument.

Contaminant reads from PhiX, the plasmids present in the assay (with the mini-CRISPR loci masked), or the *E. coli* genomes were removed by mapping the raw fastq reads to the respective elements with BowTie2.^28^ The remaining paired-end reads were merged using paired-end read merger (PEAR).^29^ Sample barcodes (included in the primers used to generate the expanded array amplicons) and expected sequence features (e.g., CRISPR repeats, existing spacers, and the region downstream of the CRISPR loci) were identified in each read by string-matching with a table of reference features. Reads with matching forward and reverse sample barcode pairs were then filtered to remove unexpanded arrays or arrays with spacers less than 20 bp or greater than 60 bp.

Protospacer sequences in the target plasmids and *E. coli* were identified by mapping the spacer sequences using GASSST^30^ with the following parameters: percentage match of 85, word size of 7, sensitivity of 5, and no gaps allowed. Spacer–protospacer hits were subsequently filtered for perfect spacer–protospacer matches (no mismatches). Sequence motif (PAM) enrichment scores were analyzed using EDLogo^31^ and were displayed using ggseqlogo.^32^

## Results

### Cas4 and Cas12 are not essential for spacer acquisition

**FIG. 1.**
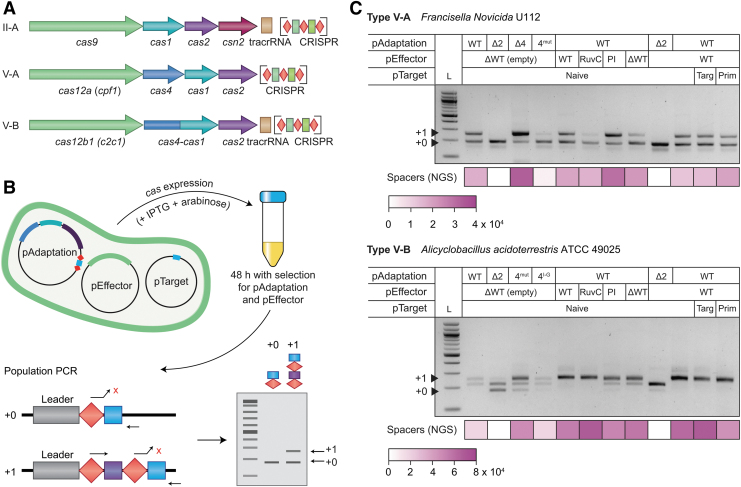
Cas4 and Cas12 are not required for adaptation of type V-A and V-B systems. **(A)** Schematic of the CRISPR-Cas loci for the type II-A, V-A, and V-B systems. The CRISPR arrays (enlarged for clarity) consist of repeats (red diamond) and spacers (*green*). **(B)** Workflow schematic of the adaptation assay using the three-plasmid system (pAdaptation, pEffector, and pTarget) in *Escherichia coli*. l-Arabinose and IPTG are added to induce expression of *cas* genes. Cells are grown for 48 h in a medium with selection for pAdaptation and pEffector, but not pTarget, and subsequently used in a population PCR to detect new spacers. The forward PCR primer matches the repeat plus a single 3′ nucleotide that mismatches with the first base of the existing spacer, allowing preferential amplification of expanded (+1) compared with unexpanded (+0) arrays.^27^ Amplified CRISPR arrays are visualized on an agarose gel electrophoresis. **(C)** Population PCR of cells expressing type V-A or V-B *cas* genes (and variations thereof). CRISPR-arrays were amplified and visualized by agarose gel electrophoresis. Plasmid variants are indicated above the gel. pAdaptation WT = Cas4, Cas1, and Cas2 (V-A) or Cas4/1 and Cas2 (V-B), Δ2 = ΔCas2, Δ4 = ΔCas4, 4^mut^ = mutated Cas4. Cas4^I-G^ = wild-type Cas4 domain swapped with Cas4 domain of type I-G from *Geobacter sulfurreducens*. pEffector WT = Cas12a/b, ΔWT = ΔCas12a/b, RuvC = catalytically inactive Cas12a/b, PI = Cas12a/b mutated in the PI domain. pTarget: Naive = pNaive, Targ = pTargeted (with protospacer and PAM), Prim = pPriming (protospacer containing a mismatch in the seed position 1). Amplicons from expanded arrays containing one new spacer (+1 arrays) are indicated by a *black triangle*. In addition to the +1 band found in V-B, an approximately +1/2 band was also observed, which sequencing revealed to be a PCR artifact. Heatmaps below each gel indicate the mean number of new (total) spacers detected by high-throughput sequencing of the PCR amplicons. Note, that the PCR approach used to detect spacers dictates that comparisons between samples are predominantly qualitative, whereas quantitative analyses of spacer characteristics can be performed within sample data sets. These heatmaps are intended to illustrate sufficient read depth for further analyses. All experiments were performed using three biological replicates: additional data are displayed in Supplementary Figure S3. CRISPR, clustered regularly interspaced short palindromic repeats; IPTG, isopropyl β-d-1-thiogalactopyranoside; PAM, protospacer adjacent motif; PCR, polymerase chain reaction; PI, PAM-interacting; WT, wild type.

To determine which genes are involved in adaptation, the type V-A and V-B CRISPR-Cas loci were heterologously expressed in *E. coli* using a three-plasmid setup (Fig. 1A, B), consisting of pAdaptation, pEffector, and pTarget. pAdaptation expressed an adaptation module containing either *cas4*, *cas1*, and *cas2* (type V-A) or the *cas4/1* fusion and *cas2* (type V-B), and a minimalistic CRISPR array that comprised a leader-repeat-spacer-repeat. Based on comparison to closely related Cas4 sequences, both native hosts of these systems (type V-A from *F. novicida U112* and type V-B from *A. acidoterrestris* ATCC 49025) appeared to contain genomic mutations altering the N-terminus of Cas4 (Supplementary Fig. S1).

Therefore, both *cas4* genes were corrected during cloning (V-A: 1 nt substitution, in codon-6: TAG [stop] > TTC [Leu], V-B: 1 nt insertion in codon-21/22: ATC-ATG [Ile-Met] > ATG-CAC [Met-His] to restore the reading frames) (Supplementary Fig. S1 and Methods section). pEffector expressed Cas12a or Cas12b for V-A and V-B, respectively. The third plasmid, pTarget, was used to mimic an invading MGE and was tested with three pTarget variants: pNaive (without a protospacer matching the CRISPR spacer), pTargeted (containing a protospacer and canonical PAM), and pPriming (containing a protospacer and canonical PAM but with a single base mutation in the first nucleotide of the seed region). Variations of the pAdaptation and pEffector plasmids were used to examine the *cas* gene requirements for CRISPR adaptation (Supplementary Tables S1 and S3).

Cells containing all three plasmids were grown in liquid media with inducers for *cas* gene expression and antibiotics selecting for pAdaptation and pEffector, but not for pTarget (Fig. 1B). After 48 h, cells were harvested and used for population PCRs to amplify expanded CRISPR arrays. Expanded arrays were visualized by agarose gel electrophoresis (Fig. 1B). Adaptation was detected in all conditions (+1 bands) except for the negative controls that lacked *cas2* (Fig. 1C and Supplementary Fig. S2), demonstrating that both the systems were active for spacer acquisition in *E. coli*.

To determine whether Cas4 was involved in adaptation, *cas4* was either deleted (Δ4) or made catalytically inactive (4^mut^) through an active-site point mutation (V-A: K70A; V-B: K81A).^33^ In addition, we hypothesized that prespacer substrates generated by the type I-G Cas4 domain would be compatible with the type V-B adaptation complex, since the type V-B Cas4 domain is closely related to that of the type I-G Cas4/1 fusion^34,35^ and the *Geobacter sulfurreducens* PCA type I-G PAM is similar to the *A. acidoterrestris* type V-B PAM.^36^ To test this, we included a plasmid encoding for a chimeric Cas4 protein, where the Cas4 domain of the type V-B Cas4/1 fusion was replaced by the type I-G *G. sulfurreducens* Cas4 domain. In type V-A, new spacers were acquired in the absence of Cas12a and Cas4, indicating that only Cas1 and Cas2 are essential for adaptation.

In type V-B, new spacers were acquired in the absence of Cas12b and with the Cas4 mutant, demonstrating that Cas12b and the activity of Cas4 are dispensable for adaptation (Fig. 1C and Supplementary Fig. S3). To investigate the role of the different catalytic domains in the effector nuclease, Cas12a/b were mutated in the RuvC domain (V-A: D917A, E1006A; V-B: E848A, D977A) or the PAM interaction (PI) domain (V-A: K613A, K617A; V-B: R122A, G143P).^37,38^ Consistent with the nonessential role of Cas12, mutations in the Cas12 RuvC or PI domains did not prevent adaptation in either system (Fig. 1C and Supplementary Fig. S3). Overall, new spacers were acquired in all samples containing at least Cas1 and Cas2.

### Cas4 but not Cas12 is required for PAM selection

**FIG. 2.**
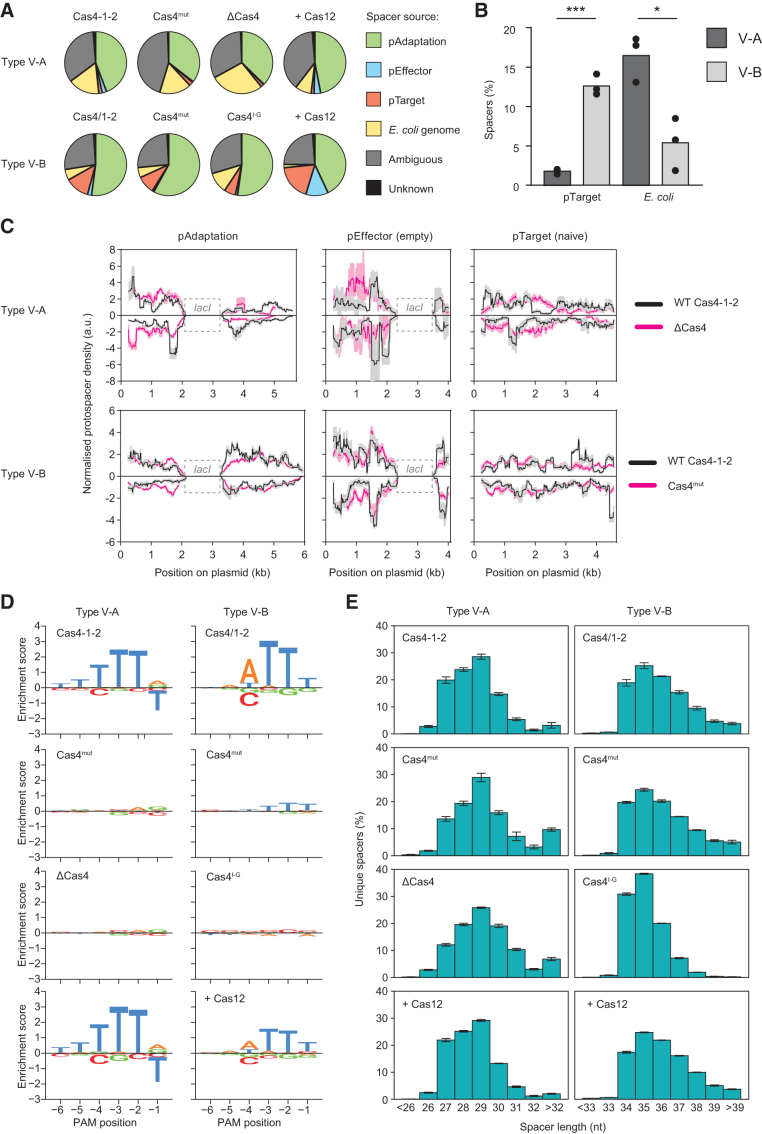
Cas4 is required for PAM selection in type V-A and V-B systems and spacer trimming in type V-A. **(A)** The relative proportions of spacers acquired from each of the plasmids and *Escherichia coli* genome. Data represent the mean of three replicates. The pAdaptation, pEffector, and the *E. coli* genome contain identical copies of *lacI*, which resulted in some ambiguous protospacers whose origins we could not differentiate (*gray*). Cas4-1-2 = Cas4, Cas1, and Cas2 (V-A) or Cas4/1 and Cas2 (V-B). Cas4^mut^ is Cas4-1-2 containing a catalytically inactive Cas4. ΔCas4 = Cas1 and Cas2 (V-A). Cas4^I-G^ = Cas4 domain of type I-G fused to Cas1 and Cas2 (V-B). +Cas12 = Cas4-1-2 and Cas12a/b. In conditions lacking Cas12, an empty plasmid or backbone was used instead. **(B)** The bias toward adaptation from foreign elements (pTarget) versus the host genome (*E. coli*) differed between the WT type V-A and V-B samples. Data represent the mean of three replicates, and statistical significance was tested using an unpaired two-sided *t*-test. ***p < 0.001 or *p < 0.05. **(C)** The locations of spacers acquired from each of the three plasmids present in the WT (*black lines*) and Cas4 deletion or mutant samples (*magenta lines*). Data were smoothed with a sliding window with a width of 250 bp and represent the mean (*solid lines*) ± SEM (shaded) for three replicates. Protospacers mapping to the forward or reverse strands are plotted above or below the x axis, respectively. The *lacI* regions (*dashed boxes*) that are shared between pAdaptation, pEffector, and the *E. coli* genome were excluded from the mapping analysis. **(D)** Sequence motif preferences for the 5′-PAM positions −6 to −1 for type V-A and V-B, based on unique spacers; the consensus interference-proficient PAMs for these systems are TTTV and NTTN, respectively.^39,40^ The enrichment and depletion scores were generated using EDLogo.^31^ **(E)** Histogram of unique spacer lengths for type V-A and V-B. Error bars represent mean ± SEM and were calculated using biological replicates (*n* = 3). SEM, standard error of the mean.

Next, we investigated whether Cas4 or Cas12 was involved in the generation, selection, or processing of prespacer substrates. For each expanded CRISPR-array sequenced, we identified the corresponding protospacers by mapping the acquired spacer sequences to the plasmids included in each sample, and to the *E. coli* genome (Fig. 2A). pAdaptation, pEffector, and *E. coli* each possesses identical *lacI* genes, which resulted in some ambiguous protospacers, the origins of which could not be identified, and so, these were excluded from further analyses. For both systems, the proportion of spacers acquired from the *E. coli* chromosome was reduced when Cas12 was present, likely due to lethal self-targeting. Also, differences were observed in the relative amounts of spacers acquired from the three plasmids for both type V-A and type V-B systems. For example, pTarget was a relatively inefficient source of spacers for type V-A (Fig. 2B).

These data suggest the underlying differences in Cas-dependent prespacer generation or selection between the two systems. Indeed, the locations of protospacers acquired from each plasmid differed in the presence and absence of Cas4 and between the type V-A and V-B WT (wild-type) samples (Fig. 2C). Next, we examined whether the observed differences in protospacer source resulted from the selection of spacers with different PAMs. For the wild-type type V-A and V-B samples, we observed enrichment for 5′-TTTV and 5′-ATTN PAMs, respectively (Fig. 2D and Supplementary Fig. S4). There were no sequence preferences downstream of the protospacers (3′-adjacent) for either system (Supplementary Fig. S5). The 5′ PAMs are consistent with the reported interference-proficient PAMs for FnCas12a and AaCas12b (5′-TTTV and 5′-NTTN, respectively).^39,40^ When Cas4 is either knocked out or made catalytically inactive, canonical PAM selectivity was largely abrogated, with the exception being a small enrichment for 5′-NTTT with the type V-B Cas4^mut^ (Fig. 2D).

This low-level enrichment might be due to PAM specificity by the type V-B Cas1. By contrast, Cas12a/b did not appear to influence PAM selection (neither did the dCas12 or PAM-interacting domain mutants, Supplementary Fig. S6), indicating that Cas12 is not involved in PAM selection in either type V-A or V-B.

In class 1 CRISPR-Cas systems, Cas4 directly (via endonucleolytic cleavage of the PAM-proximal end) and indirectly (by “shielding” this end from trimming by host exonucleases) effects trimming of prespacers, and hence influences the lengths of acquired spacers.^14,15,33,41^ For the wild-type (Cas4-1-2) type V-A system, we observed a distribution of spacer lengths with the modus (most frequently observed spacer length) at 29 nt, with more shorter than longer spacers (Fig. 2E and Supplementary Fig. S7A). The mutation (Cas4^mut^) or removal (ΔCas4) of Cas4 resulted in a change in the spacer length distribution toward longer spacers compared with the wild type (Cas4-1-2): still a modus of 29 nt, but with more longer than shorter spacers (Fig. 2E and Supplementary Fig. S7A) (comparison of fractions of fragments larger than the mode, after one-way ANOVA, using *t*-test with Dunnett correction of *p*-values: comparing Cas4^mut^ and wild type: *p* = 0.0038, and comparing ΔCas4 and wild type: *p* = 0.017).

For type V-B, the spacer length distribution was less affected by the Cas4 point mutation (Cas4^mut^), with the modus at 35 nt and more longer than shorter spacers, similar to the wild type (Fig. 2E and Supplementary Fig. S7B). However, swapping the Cas4 domain of the type V-B Cas4/1 fusion with a Cas4 domain from an I-G system (Cas4^I-G^) resulted in a strong narrowing of the spacer length distribution (comparison of standard deviations, after one-way ANOVA, between Cas4^I-G^ and wild type: *p* = 0.0053). By contrast, in the presence of Cas12, spacer length distributions were similar to the wild type (i.e., more longer than shorter spacers, Fig. 2E and Supplementary Fig. S7B), indicating that Cas12 has no major effect on spacer length. Overall, our data demonstrate that Cas4 but not Cas12 is essential for PAM selection in type V-A and V-B systems and that Cas4 contributes to prespacer processing or trimming for type V-A.

### No evidence of primed type V adaptation

**FIG. 3.**
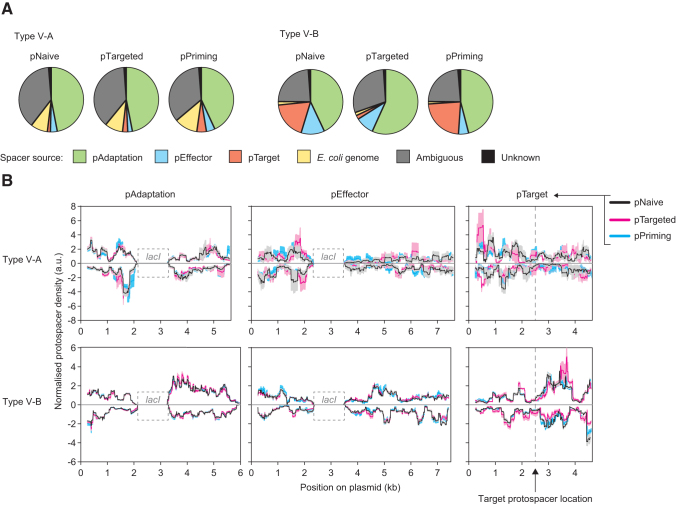
No primed adaptation in type V-A or V-B systems. **(A)** The relative proportions of spacers acquired from each of the plasmids and *Escherichia coli* genome. The data represent the mean of three replicates. pAdaptation, pEffector, and the *E. coli* chromosome contain identical copies of *lacI*, which resulted in some ambiguous protospacers whose origins we could not differentiate. **(B)** The protospacer mapping distributions to the plasmids in each sample. Data were smoothed with a 250 bp sliding window and represent the mean (*solid lines*) ± SEM (*shaded*) for either two or three replicates; for some samples with low numbers of reads, the normalized mapping for one of the three replicates contained outlying data points, and so, in these cases, where the other two replicates were in close agreement, the outlying replicate was excluded from the analyses. Samples contained the WT pAdaptation and pEffector (+Cas12) plasmids with different versions of pTarget: pNaive (*black*), pTargeted (*magenta*), or pPriming (*cyan*). Protospacers mapping to the forward or reverse strands are plotted above or below the x axis, respectively. The *lacI* regions (dashed boxes) that are shared between pAdaptation, pEffector, and the *E. coli* genome were excluded from the mapping analysis.

Some CRISPR-Cas systems exploit target recognition by existing spacers to stimulate the acquisition of additional spacers through a process termed primed CRISPR adaptation (priming).^2,3^ Based on theoretical and observed protospacer mapping for primed adaptation in other CRISPR-Cas types, for example, I-B, I-E, I-F, and II-A,^4,7,42–45^ we expected that priming in type V would result in an increased abundance of spacers acquired from pTarget and possibly in close proximity to the site targeted by the existing spacer (the target protospacer location). To test whether priming occurs in type V-A and V-B, we included samples with the WT pAdaptation and pEffector (Cas12) plasmids in combination with the pNaive, pTargeted, or pPriming variants of pTarget (Figs. 1C and 3A).

The protospacer mapping distributions that we observed for pTarget in the type V-A and V-B target (pTargeted) and priming (pPriming) samples were not notably different to the corresponding samples containing untargeted (pNaive) plasmids (Fig. 3B). For the type V-B system, we observed a decrease in the total number of spacers acquired from pTarget, which is consistent with interference-mediated cleavages of the plasmid and thereby eliminating pTarget as a source of potential adaptation. As observed in the absence of Cas12 (Fig. 2C), the WT type V-A and V-B systems displayed distinct mapping patterns across all the plasmids (Fig. 3B, black lines), indicative of different prespacer generation or selection mechanisms for the type V-A versus the type V-B system. Overall, using our experimental setup, we did not observe evidence of primed adaptation for either the type V-A or V-B systems.

### Naive adaptation by type V-A and V-B systems is biased toward locations of RecBCD activity

**FIG. 4.**
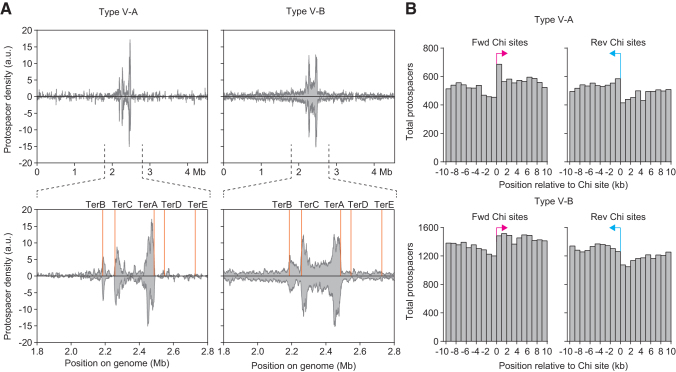
Naive adaptation is biased toward locations of RecBCD activity. **(A)** Spacers acquired from the host chromosome for the WT Cas4-1-2 samples. The *Escherichia coli* genome is displayed linearized starting at the OriC, and spacers mapping to *lacI* were excluded. Data represent the median for six replicates and are smoothed with a rolling-sum window of 10 kb, and then normalized to the total number of spacers in each sample. **(B)** The distribution of protospacers around Chi sites in the *E. coli* genome, with the sum of all spacers acquired across six replicates binned into 1 kb sections, as indicated. Protospacers and Chi sites within the genome terminus region (2.0–2.6 Mb) were excluded from this analysis. Note, although the total number of spacers detected was higher for type V-B than type V-A (due to differences in sequencing depth), the type V-A system acquired a higher proportion of all spacers from *E. coli*, relative to other sources, than type V-B (Fig. 2A).

Despite the absence of evidence supporting primed CRISPR adaptation, we did observe clear differences in the naive adaptation preferences between type V-A and V-B (Figs. 2A and 3A). In the absence of Cas12 (eliminating the possibility of lethal self-targeting the *E. coli* genome), the type V-A system acquired >10-fold more spacers from the *E. coli* genome relative to pTarget than the type V-B system (Fig. 2B). To further investigate this effect, we analyzed the distribution of protospacer locations within the *E. coli* genome (Fig. 4). For both type V-A and V-B, most host-derived spacers were acquired from the *E. coli* genome terminus region, with increases in protospacer density immediately adjacent to TerA, TerB, and TerC (Fig. 4A).^46^

A previous study of naive adaptation by the *E. coli* type I-E CRISPR-Cas system revealed enrichment for spacers acquired from regions that are expected to be associated with high RecBCD activity, including regions adjacent to Chi sites.^25^ We observed similar asymmetric spacer acquisition relative to Chi sites, with more spacers acquired downstream of Chi sites (relative to the Chi site orientation) (Fig. 4B). These data are consistent with RecBCD-stimulated CRISPR adaptation at DNA breaks.^25,27^

## Discussion

In this study, we investigated the Cas protein requirements for naive adaptation for the type V-A and type V-B systems from *F. novicida* and *A. acidoterrestris*, respectively. For both systems, we found that although Cas1 and Cas2 are necessary and sufficient for spacer acquisition, the ancillary protein Cas4 is required for correct PAM selection. We noted that the loss of Cas4 (ΔCas4) appeared to stimulate spacer acquisition rates when compared with WT and Cas4^mut^. This might indicate that sequestering the PAM-end of the acquired spacer by a catalytically inactive Cas4 obstructs spacer integration, leading to lower adaptation rates.^14,41^ Cas4-dependent PAM selection has been previously reported in several type I systems.^14–16,41^ In addition, the *Pyrococcus furiosus* Cas4-2 (one of two Cas4s encoded by this host containing type I-A, I-B, and III-B systems) controls directional spacer integration, as a Cas4-2 knockout resulted in canonical PAMs being present on both ends of the protospacers.^15^

We did not observe any evidence for a similar motif here for either type V-A or V-B (Supplementary Fig. S5). Within the type V family, types V-C and V-D only contain Cas1, which in tetramer form has been shown to be sufficient for type V-C adaptation.^23^ Interestingly, other type V subtypes do not contain either Cas1 or Cas2 and although it remains unclear how these systems acquire new spacers against new incoming MGEs, it seems most likely that co-occurring CRISPR-Cas systems provide this function.^24,35,47^

For type II-A, the effector nuclease Cas9 and the ancillary protein Csn2 are required for correct PAM selection.^17,18^ In contrast, we did not find any influence of Cas12a or Cas12b on PAM selection. Our swap of the type V-B Cas4/1 Cas4 domain, with that of the closely related type I-G Cas4/1 fusion, did not acquire spacers with correct PAMs. However, the Cas4^I-G^/1^V-B^ fusion did narrow the length distribution of spacers acquired, thereby demonstrating the influence of this chimeric protein on spacer trimming. By contrast, mutational inactivation of the native type V-B Cas4 domain did not markedly influence spacer length. Cas4 mutation in type V-A did result in an increase in longer spacers (Fig. 2E). The different effect of Cas4 activity between these systems might relate to the presence of the Cas4/Cas1 fusion protein, since similar effects were observed for type I-G (fused Cas4/1 and no spacer length effect) and type I-D (single Cas4 and there was a spacer length effect).^16,36^

These changes in spacer length might be due to the structural differences of the adaptation complex caused by the ratio of Cas4 and Cas1, which exists in equimolar in a fusion protein, but can differ in an unfused system.^41^ For example, in type I-C and I-D, one Cas4 protein interacts with a Cas1 dimer.^14,48^ Therefore, the spacer trimming effects might be specific to systems with unfused, stand-alone Cas4 proteins. However, even though the effects of spacer trimming by Cas4/1 from V-B was not observed in this study, we cannot exclude a role for Cas4/1 in prespacer processing, as observed in other Cas4/1 systems.^41^ Overall, we found Cas4 to be essential for the acquisition of interference-proficient spacers in both types V-A and V-B, but the role of Cas4 in spacer processing likely differs between the systems due to different ruler and/or processing mechanisms.

In addition to differences in PAMs and spacer lengths between type V-A and V-B, the systems displayed different preferences for the source of new spacers. For both systems, the majority of spacers acquired were derived from pAdaptation, which has the highest copy number (∼20–40) compared with pEffector (∼10–12 copies) and pTarget (∼15–20 copies). A similarly high proportion of spacers derived from pAdaptation was found in type I-G, where the CRISPR locus was overexpressed using the same plasmids used in this study.^36^ However, it is unlikely that the copy number is the primary determinant for this bias, as the other two plasmids only marginally deviate from this. Moreover, the type V-A system acquired very few spacers from pTarget or pEffector, whereas pTarget was a relatively good source of spacers for the type V-B system (Fig. 2A).

The relative proportions of spacers acquired from the *E. coli* genome also differed substantially between the type V-A and V-B systems. Indeed, the balance between acquisition from episomal MGEs versus the host chromosome seems to differ markedly between the CRISPR-Cas types. For example, in types I-D and II-A, most spacers were acquired from the chromosome instead of plasmids.^16,18^ Acquisition of spacers from the host chromosome is typically detrimental, due to self-targeting and cell death, or loss of CRISPR-Cas systems.^7,49–51^ As such, mechanisms that improve the bias of spacer acquisition toward foreign genetic material are likely beneficial.

A previous study on adaptation in type I-E showed that due to typically higher copy numbers and more rapid replication of MGEs than the host chromosome, stalled replication forks occur more frequently on plasmids, which leads to RecBCD-mediated generation of DNA fragments that are suitable prespacer substrates.^25,52^ We also observed hotspots for adaptation from the host chromosome replication termination (Ter) sites, where replication fork stalling, and hence, RecBCD or AddAB activity, is more common.^43^ However, we observed that type V-A and V-B differed in the relative amounts of spacers acquired from near Ter sites compared with elsewhere in the genome (Fig. 4).

The most likely mechanistic explanations for the differences between the sources of spacers for type V-A versus V-B are differences in either prespacer generation or prespacer compatibility and selection by the adaptation machinery. For example, the DNA fragments produced by RecBCD might be more compatible with type V-A than V-B, perhaps due to differences in the fragment length of the proximity of PAM sequences near the ends of fragments.^53,54^ For primed adaptation in type I systems, the Cas3 nuclease generates prespacer fragments enriched for PAMs near their 3′ ends, which increases the efficiency of adaptation.^6,52^ Since the ends of DNA fragments produced by RecBCD are not specifically enriched for type V-A- or V-B-compatible PAMs, very few fragments might natively be good prespacer substrates.

Intriguingly, the native hosts of the type V systems studied here appear to contain deleterious mutations in *cas4* and *cas4-1* (Supplementary Fig. S1). According to our results, loss of Cas4 activity would result in loss of PAM selection in these systems, requiring natural selective processes (such as a host surviving phage infection due to acquisition of an interference-capable spacer) to select for PAM-proficient spacers. *In vivo* spacer acquisition experiments with the native hosts are needed investigate the biological implications of these mutations. Overall, our findings demonstrate that several mechanisms of CRISPR adaptation are conserved between diverse CRISPR-Cas systems, including the influence of Cas4 on PAM selection and spacer trimming, and acquisition hotspots from RecBCD-derived prespacer substrates. However, we also found that subtle variations between closely related systems, such as types V-A and V-B, can substantially affect the bias of immunity toward foreign genetic elements.

## Supplementary Material

Supplemental data

Supplemental data

Supplemental data

Supplemental data

Supplemental data

Supplemental data

Supplemental data

Supplemental data

Supplemental data
