# Supplementary material for: Adaptation by Type V-A and V-B CRISPR-Cas Systems Demonstrates Conserved Protospacer Selection Mechanisms Between Diverse CRISPR-Cas Types
Source: CRISPR J. 2022 Aug 12;5(4):536–47. doi: 10.1089/crispr.2021.0150 (PMC9419969; doi:10.1089/crispr.2021.0150)
Supplement: Supplemental data [file Suppl_FigS1.docx]

**Figure S1: Fixing the Cas4 by multiple alignment.** Amino acid sequences were obtained from UniProt and multiple sequence alignment was done using ClustalO. (A) Multiple alignment using Cas4 from various type V-A systems. Black line indicates the Cas4 protein used in this study and red arrow indicates a premature stop codon due to a nonsense mutation. Cas4 proteins use for this alignment are PcCas4, AiCas4, MoCaS4, FsCas4, MiCas4 and FnCAs4 from *Porphyromonas crevioricanis*, *Acinetobacter indicus*, *Moraxella* sp. VT-16-12, *Fibrobacter succinogenes*, *Moraxella lacunata* and *Francisella Novicida*, respectively. **(B)** Multiple alignment using Cas4 from various type V-B systems. Black line indicates the Cas4 protein used in this study and red arrow indicates a frameshift at the N-terminal-end due to a deletion causing a frameshift. Cas4/1 proteins used for this alignment are AkCas4, CsCas4, BpCaS4, BaCas4, BhCas4 and AaCas2 from *Alicyclobacillus kakegawensis*, *Candidatus Sulfopaludibacter*, *Brevibacillus parabrevis*, *Bacillus* sp. SYSU G01002, *Bacillus hisashii*, and *Alicyclobacillus acidoterrestris* respectively. **(C**) DNA and amino acid sequence of corrected FnCas4 for type V-A. An A to T mutation changes a premature stop codon to a leucine (L). **(D)** DNA and amino acid sequenced of corrected AaCas4 for type V-B. A G insertion causes a frameshift and restores the correct open reading frame.
