# Supplementary material for: Adaptation by Type V-A and V-B CRISPR-Cas Systems Demonstrates Conserved Protospacer Selection Mechanisms Between Diverse CRISPR-Cas Types
Source: CRISPR J. 2022 Aug 12;5(4):536–47. doi: 10.1089/crispr.2021.0150 (PMC9419969; doi:10.1089/crispr.2021.0150)
Supplement: Supplemental data [file Suppl_FigS2.docx]

**Figure S2: CRISPR-array amplification using unmodified and 3’ phosphorothioate modified primers.** Amplification of the CRISPR-array was done using anneal temperature ranging from 50-70 °C. Expanded and non-expanded CRISPR-array are indicated by a +1 and +0 arrow, respectively.
