# Supplementary material for: Adaptation by Type V-A and V-B CRISPR-Cas Systems Demonstrates Conserved Protospacer Selection Mechanisms Between Diverse CRISPR-Cas Types
Source: CRISPR J. 2022 Aug 12;5(4):536–47. doi: 10.1089/crispr.2021.0150 (PMC9419969; doi:10.1089/crispr.2021.0150)
Supplement: Supplemental data [file Suppl_FigS3.docx]

**Figure S3: Replicate gel images for Figure 1.** Population PCR of cells expressing type V-A or V-B cas genes (and variations thereof). CRISPR-arrays were amplified and visualised by agarose gel electrophoresis. Plasmid variants are indicated above the gel. pAdaptation WT = Cas4, Cas1 and Cas2 (V-A) or Cas4/1 and Cas2 (V-B), Δ2 = ΔCas2, Δ4 = ΔCas4, 4mut = mutated Cas4. pEffector WT = Cas12a/b, ΔWT = ΔCas12a/b, RuvC = catalytically inactive Cas12a/b, PI = Cas12a/b mutated in the PAM-interacting (PI) domain. pTarget: Naive = pNaive, Targ = pTargeted (with protospacer and PAM), Prim = pPriming (protospacer containing a mismatch in the seed position 1).
