# Supplementary material for: Adaptation by Type V-A and V-B CRISPR-Cas Systems Demonstrates Conserved Protospacer Selection Mechanisms Between Diverse CRISPR-Cas Types
Source: CRISPR J. 2022 Aug 12;5(4):536–47. doi: 10.1089/crispr.2021.0150 (PMC9419969; doi:10.1089/crispr.2021.0150)
Supplement: Supplemental data [file Suppl_FigS4.docx]

**Figure S4: PAM frequencies displayed as k-mers for the 5ʹ-PAM positions.** Data represent the mean of three replicates.
