# Supplementary material for: Adaptation by Type V-A and V-B CRISPR-Cas Systems Demonstrates Conserved Protospacer Selection Mechanisms Between Diverse CRISPR-Cas Types
Source: CRISPR J. 2022 Aug 12;5(4):536–47. doi: 10.1089/crispr.2021.0150 (PMC9419969; doi:10.1089/crispr.2021.0150)
Supplement: Supplemental data [file Suppl_FigS6.docx]

**Figure S6: The 5ʹ-PAM preferences observed in the dCas12 and Cas12 PAM-interacting (PI) domain mutants.** Data represent the mean of three replicates. The enrichment and depletion scores were generated using EDLogo and displayed using ggseqlogo.^31, 32^
