# Supplementary material for: Adaptation by Type V-A and V-B CRISPR-Cas Systems Demonstrates Conserved Protospacer Selection Mechanisms Between Diverse CRISPR-Cas Types
Source: CRISPR J. 2022 Aug 12;5(4):536–47. doi: 10.1089/crispr.2021.0150 (PMC9419969; doi:10.1089/crispr.2021.0150)
Supplement: Supplemental data [file Suppl_FigS7.docx]

**Figure S7: Percentage of spacer shorter or longer than the modus. (A)** V-A modus = 29 nt and **(B)** V-B modus= 35 nt. Data represent the mean of three replicates.
